# Microfluidic Biosensors for the Detection of Motile Plant Zoospores

**DOI:** 10.3390/bios15030131

**Published:** 2025-02-21

**Authors:** Peikai Zhang, David E. Williams, Logan Stephens, Robert Helps, Irene Patricia Shamini Pushparajah, Jadranka Travas-Sejdic, Marion Wood

**Affiliations:** 1 Centre for Innovative Materials for Health, School of Chemical Sciences, The University of Auckland, Auckland 1010, New Zealand; pzha503@aucklanduni.ac.nz; 2 Auckland Bioengineering Institute, The University of Auckland, Auckland 1010, New Zealand; 3 MacDiarmid Institute for Advanced Materials and Nanotechnology, Wellington 6140, New Zealand; 4 inFact Limited, Christchurch 8013, New Zealand; logan.stephens@infact.co.nz (L.S.); robert@infact.co.nz (R.H.); 5 The New Zealand Institute for Plant & Food Research Limited, Auckland 1025, New Zealand; shamini.pushparajah@plantandfood.co.nz

**Keywords:** microfluidic device, electrochemical biosensor, zoospore detection, chemotaxis, multi-physics simulation

## Abstract

Plant pathogen zoospores play a vital role in the transmission of several significant plant diseases, with their early detection being important for effective pathogen management. Current methods for pathogen detection involve labour-intensive specimen collection and laboratory testing, lacking real-time feedback capabilities. Methods that can be deployed in the field and remotely addressed are required. In this study, we have developed an innovative zoospore-sensing device by combining a microfluidic sampling system with a microfluidic cytometer and incorporating a chemotactic response as a means to selectively detect motile spores. Spores of *Phytophthora cactorum* were guided to swim up a detection channel following a gradient of attractant. They were then detected by a transient change in impedance when they passed between a pair of electrodes. Single-zoospore detection was demonstrated with signal-to-noise ratios of ~17 when a carrying flow was used and ~5.9 when the zoospores were induced to swim into the channel following the gradient of the attractants. This work provides an innovative solution for the selective, sensitive and real-time detection of motile zoospores. It has great potential to be further developed into a portable, remotely addressable, low-cost sensing system, offering an important tool for field pathogen real-time detection applications.

## 1. Introduction

Zoospores are motile spores produced by certain types of fungi, bacteria, protists and oomycetes that swim by using their flagella with a speed of up to 250 µm/s [1]. Plant pathogen zoospores play a key role in spreading a number of serious plant diseases, causing massive ecological and economic losses worldwide [2]. Even today, Potato Late Blight (PLB) caused by *Phytophthora infestans* continues to be the foremost biotic threat to worldwide potato production, posing a significant menace to global food security, particularly in less developed regions reliant on potatoes as a primary food source [3,4]. Worldwide, the economic impact of yield loss and management expenses due to PLB can range from USD 3 to 10 billion annually [4,5]. Another specific and distressingly impactful example is Kauri Dieback, caused by *Phytophthora agathidicida.* It is a devastating disease leading to decline and mortality in kauri trees (*Agathis australis*) across northern New Zealand. These trees are among the longest-living species globally and hold significant cultural importance, particularly among Māori indigenous people.

**Figure 1:**
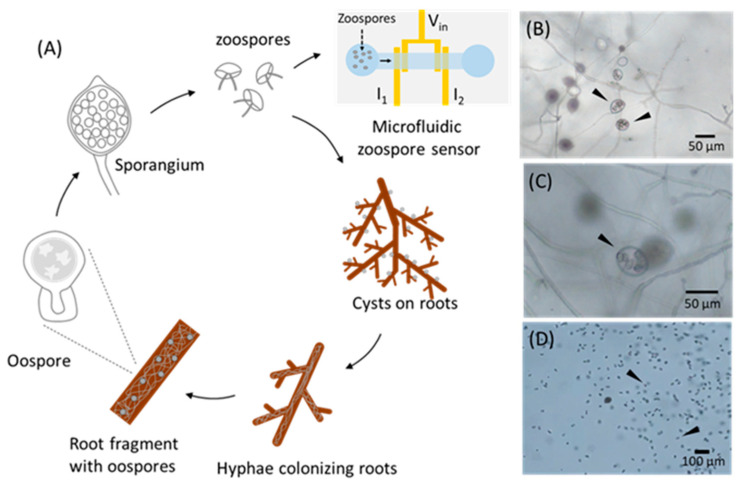
(**A**) Schematic illustration of life cycle of zoospores. (**B**–**D**) Optical microscope images of *Phytophthora cactorum* H78. (**B**) Mature sporangia harbouring zoospores (►). (**C**) Individual sporangium highlighting membrane-engaged zoospores prior to release (►). (**D**) Motile zoospores released from sporangia into water (►).

The infection of Kauri occurs when oospores of *P. agathidicida* in soil or plant debris germinate, producing sporangia, which then release motile asexual zoospores into the immediate environment (Figure 1). These zoospores subsequently infect the sensitive, feeder roots of kauri trees, eventually spreading to the main roots and lower trunk. Once the lower trunk is affected, bleeding lesions or cankers develop, progressively encircling the trunk and leading to girdling and eventual tree demise [1]. There is as yet no established treatment for infected trees and most kauri trees infected with *P. agathidicida* will ultimately perish. Therefore, the real-time detection of zoospores is critical not only to determining the presence and subsequent management of the pathogen but also to understanding their physiology, such as how zoospores interact with the local microenvironment, what their distribution pattern is and how they communicate with each other.

Traditional methods used for detecting zoospores include microscopic observation, molecular techniques such as qPCR and immunological methods. Microscopic observation involves techniques like soil baiting and soil or water sample collection [6,7,8]. However, the small size (8 to 12 µm) and high mobility of zoospores make microscopic observation highly time- and labour-consuming. In addition, the concentration of zoospores can be very low in the field, especially during the early infection stages, which brings challenges to microscopic detection. Molecular techniques and immunological methods, such as quantitative polymerase chain reaction (qPCR) [9,10,11,12], enzyme-linked immunosorbent assays (ELISAs) [13] and zoospore-trapping immunoassays (ZTIs) [14,15] offer higher sensitivity and specificity. However, they still rely on the collection of samples from soil or water, which may fail to collect low-concentration zoospores. In addition, they require specialised equipment and reagents, and the accuracy of the results can be affected by inhibitors present in the samples. The comparison of different zoospore detection techniques has been included in several reviews [7,14,16,17].

In recent years, microfluidic-based flow cytometry sensors have been developed for cell counting [18,19,20,21,22,23,24,25,26,27,28,29,30,31]. Their core part is a microchannel with paired microelectrodes, normally pre-patterned on the substrate, inside the channel [27,32,33]. The passing of cells driven by a syringe pump triggers a change in impedance measured between the two electrodes [28], as has been analysed in detail by Sun and Morgan [29].

However, these studies are mainly limited to non-motile cells and polymeric beads and have not been used for motile targets, such as zoospores. Importantly, as the targets are typically carried by a flow driven by a syringe pump, one significant limitation associated with this is the non-specificity. When using such platforms for zoospore sensing, a variety of micro-objects, such as dust particles, microfibres and other small organisms, can be carried by the flow, be non-specifically introduced into the sensing channel and trigger an impedance change. Several strategies can be used to minimise non-specific sensing signals, including a pre-purification step of the sampling solution, the flow rate-controlled separation of particles based on their physical properties (such as density difference) and signal filtration based on the different signatures, such as the amplitude and duration of the peak, recorded from different targets. These strategies minimise the detection of fault signals; however, differentiating signals from targets of similar impedance and physical properties, such as between different zoospore species or between encysted vs. non-encysted zoospores, is still challenging.

Zoospores are known to be responsive to several environmental factors, including chemical factors, pH, oxygen gradients and electrical fields [1]. Therefore, instead of using a carrying flow for the passive presence of zoospores, a promising strategy is to exploit the inherent biological response of the pathogen itself and use an attractant to direct the actively swimming zoospores into the sensing device.

In this work, we developed a new microfluidic zoospore sensor based on the chemotaxis behaviour of zoospores and the impedance change between paired electrodes. *Phytophthora cactorum* zoospores (Figure 1B–D) were used as the model zoospores. By creating a gradient of chemo-attractants, the zoospores could be selectively guided to swim into the channel and be detected electrically. After a simulation study and the optimisation of the sensing parameters, high-quality signals with a high signal-to-noise ratio were successfully recorded when the zoospores passed the electrodes. Such a platform provides a unique and highly promising solution for the real-time, selective and sensitive detection of motile zoospores. The whole sensing setup is easy to fabricate and cost-effective; thus, it has great potential to be further developed for field applications for plant pathogen management.

## 2. Materials and Methods

### 2.1. COMSOL Simulation

The simulation study was carried out by using finite element simulation software, COMSOL Multiphysics (V6.0). The size of zoospores was expected to be around 8–12 µm. In this study, we used 10 µm for the sphere models and modelled the membrane as a 0.1 µm thick dielectric membrane with relative permittivity of 1. A channel was 20 µm wide and 20 µm deep, while the Z position of the sphere centre is at 10 µm, which was in the middle of the channel selected. The electric current (EC), single-phase flow (SPF) and transport of diluted species (TDS) modules were used in these simulations.

### 2.2. Fabrication of Microfluidic Devices

Conventional photolithography methods were used for the fabrication of microfluidic devices. A schematic of the fabrication process is presented in Supplementary Materials Figure S1. Gold electrodes were patterned from Au- (100 nm) and Ti-coated (40 nm) glass substrates (Deposition Research Lab, Inc., Saint Charles, MO, USA). The width of each Au electrode was 50 µm, and the spacing between two electrodes was 10 µm. There were two pairs of sensing electrodes inside each microfluidic channel. The distance between these two pairs was 1000 µm. The fluidic channels were formed by casting poly(dimethylsiloxane) (PDMS; Sylgard 184, Dow, Midland, MI, USA) into moulds made of photoresist SU-8. The detailed fabrication parameters are included in the Supplementary Materials (Section S1, Microfluidic chip fabrication). The height and width of the microfluidic channels were characterised by an optical profilometer (ContourX-100, Bruker, Billerica, MA, USA). The photolithography technique provided high resolution and fidelity for both the PDMS channels and Au electrodes. Figure S2 in Supplementary Materials shows optical microscopy and 3D profilometer images of the fabricated electrodes and channels.

A puncher was used to create inlet and outlet holes in the PDMS piece for the later insertion of needles and the connection of tubing. After an O_2_ plasma treatment of both the Au-on-glass electrodes and the PDMS microfluidic channels, the two pieces were carefully aligned and pressed to contact. After baking at 80 °C for 5 min, permanent bonds formed between them. Needles and tubing were then attached to guide the flow in and out of the microfluidic channels.

### 2.3. Phytophthora Cactorum Zoospore Production

Many *Phytophthora* pathogens, for example, *Phytophthora agathidicida*, the causative organism of Kauri Dieback, require handling under highly restrictive containment due to their pathogenic nature. As an alternative, non-restricted model organism *Phytophthora cactorum* was used, since it displays similar size, motility and encystment behaviour.

Purified cultures were prepared on solidified nutritive agar, which was then used to infect sterilised oats. Once inoculated, these oats were placed in sterile water and incubated under light conditions at room temperature to stimulate the production of sporangia (the capsules within which the motile spores grow). The release of the zoospores was initiated following a short period of cold shock. The released zoospores were collected by using a syringe. The procedure is described in detail in Supplementary Materials File S1, Section S4 (*Phytophthora cactorum* zoospore production), and Video S1 shows the release process of zoospores. The zoospore preparations were prepared fresh on the day of experimentation and discarded after 8 h.

### 2.4. Chemotaxis Exploration

Zoospores detect the presence of root hairs by sensing and responding to a number of factors, especially chemical gradients [1]. Various *Phytophthora* species are attracted to amino acids like aspartate, glutamate, asparagine, glutamine, arginine and methionine, which are secreted by a wide range of plants [34]. *Phytophthora palmivora* is drawn to isovaleraldehyde, valeraldehyde and ante-isovaleraldehyde, which are compounds found in the root exudates of numerous plants [1], whereas *Phytophthora sojae* is attracted to isoflavones such as daidzein and genistein, which are released by soybean roots [35]. The optimisation of the chemotaxis response was initially determined in a culture dish setup and subsequently by observation in a microchannel setup in a bright-field microscope, taking care to avoid any flow through the channel. A 1% glucose solution prepared with sterile H_2_O was an effective attractant for *Phytophthora cactorum* (Supplementary Materials, Figure S3 and Video S2), with the additional advantages of ease of preparation and a low risk of triggering zoospore encystment.

### 2.5. Recording Setup

**Figure 2:**
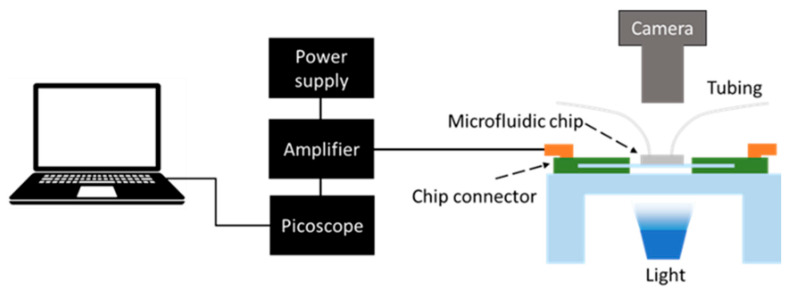
Schematic of sensing setup.

The schematic of the measurement system setup is shown in Figure 2. The in-phase magnitude of the current flow in response to AC stimulation was measured by using a current follower and single-chip lock-in amplifier (AD630EVK, inFact, Christchurch, New Zealand), which was mounted on a Printed Circuit Board (PCB) (inFact, Christchurch, New Zealand) with pogo pin connectors, against which the sensing chip was clamped. An opening in the middle of the PCB enabled the observation of the zoospores within the channel. A photo showing the PCB connector and the lock-in amplifier is presented in Supplementary Materials Figure S4. A camera with a microscope lens (Thorlabs, Newton, NJ, USA) was placed above the sensing chip, while an LED light was placed under the chip. The optical video was recorded simultaneously with the electrical recording; thus, the sensing signal could be correlated with the optical information. The lock-in amplifier output was AC-coupled to an oscilloscope (Picoscope 2000, Pico Technology, Cambridgeshire, UK) to record the time variation in impedance.

## 3. Results and Discussion

### 3.1. Design Considerations

**Figure 3:**
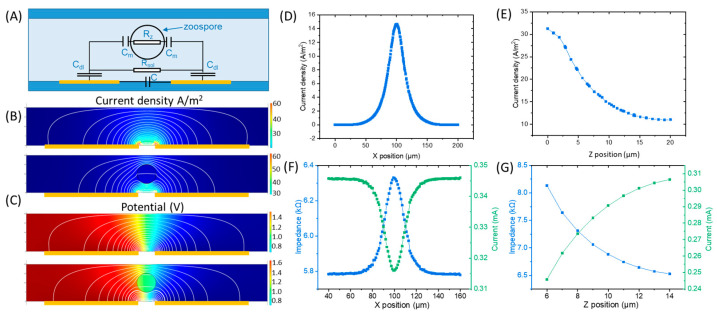
The finite element (COMSOL) simulation of the electrical field, with AC potential applied between the electrodes. The *X*-axis lies in the plane of the electrodes, and the *Z*-axis perpendicular to this (Z = 0 at the electrode surface). (**A**) A schematic of the equivalent circuit. (**B**) The distribution of the current densities at different positions in the channel (blue to red: low current density to high current density). (**C**) The distribution of the local electrical potential inside the channel (blue to red: low potential to high potential). (**D**) The calculated current density along the *X*-axis inside the channel with the Z position of zoospores at 10 µm (middle of the channel). (**E**) The calculated current density along the *Z*-axis inside the channel with the X position of zoospores at 100 µm (middle of the electrode gap). (**F**) The calculated impedance and current between the two electrodes with the zoospores at different X positions in the channel and Z = 10 µm. Blue and green lines are the impedance and current between the two electrodes, respectively. (**G**) The calculated impedance and current between the two electrodes with the zoospores at different Z positions in the channel, with X = 100 µm, the middle of the electrode gap. Blue and green lines are the impedance and current between the two electrodes, respectively.

For the design of the device, we proposed a simple cytometer concept, where zoospores would be detected by the change in electrical impedance in a microfluidic channel when a zoospore passed into the gap between the two electrodes (Figure 3A).

There are six issues to address: (1) the detection of the zoospores, determined by the minimum reliably detectable impedance change and also by the transit time of a zoospore across the electrode gap; (2) the sampling of a sufficient volume of solution, given that the zoospore concentration might be low and variable; (3) the specificity of detection, to discriminate against other particulate matter that might be present; (4) guiding or driving zoospores to enter the sensing channel, which can be achieved by using a syringe pump/chemical attractant and a suitable design of the channel shape; (5) the prevention of encystment of the zoospores in the channels, which happens if the spores are in a stressful liquid environment; and (6) the design of an instrument system that is of reasonably low cost that could be used on the field and adapted for remote measurement.

We used the measurement of the resistive (in-phase) component of the impedance with a single-chip lock-in amplifier, AC-coupled to detect current pulses associated with zoospore transit. The integration time of the lock-in amplifier determines the time response. The sensing response is highly related to the measurement frequency and channel dimensions, which will be further discussed in the later context.

Figure 3 shows the finite element computation of the current flow (Figure 3B–E) at 50 kHz and impedance |Z| (Figure 3F,G) between the electrodes, with a model zoospore passing across the 10 µm gap between electrodes disposed at the base of a channel 20 µm high and 20 µm wide. Based on the COMSOL simulation, the highest current density appeared in the gap area between the paired electrodes along the *X*-axis (Figure 3D), while along the *Z*-axis (*X*-axis in the middle of the gap), the current density increased from the top to the bottom of the channel (Figure 3E). A more prominent change in the electrical signal would be triggered when zoospores are present in the areas with higher current densities. Therefore, a higher response would be triggered when zoospores are in the middle of the gap (Figure 3F) and closer to the bottom of the channel (Figure 3G).

Finite element computation on the effect of channel and gap dimensions is presented in Supplementary Materials Figure S5. The results indicate that when the cross-sectional dimension of a channel, including height and width, is closer to the size of a zoospore, the sensing responses will be higher. To obtain a significant change (>5%) in DC resistance (R_sol_), the inter-electrode gap needed to be no more than three times and the channel height and width no more than four times the zoospore diameter.

However, constraints on the channel and electrode dimensions include whether a spore can enter and move through a channel smoothly and how challenging and repeatable the fabrication would be. For example, a narrow microchannel would suffer from blockage during sensing, and a too-small gap between Au electrodes may risk short-circuiting.

For driving zoospores into the channel, microfluidic flow cytometry arrangements described in the literature have used pumped flow. However, one significant shortcoming associated with such a setup is the lack of specificity. We have addressed the issue of the selectivity of detection by utilising the chemotaxis property of zoospores [1,36]. These spores will swim up the concentration gradient of a suitable attractant.

The device design, therefore, has the sensing electrodes disposed in a channel within which the solution is static and which connects a closed reservoir containing the attractant to the water to be sampled. A concentration gradient is established within the channel, and there is an additional gradient extending from the mouth of the channel to the water to be sampled. With no flow and a clean solution in the sensing channel, the only objects moving past the electrodes should be swimming zoospores. However, the device needs to continuously sample from the environment, implying a flow through the device and hence a transition zone where zoospores being carried by the flow could be detected and swim into the gradient of the attractant within a static zone.

It is worth mentioning that zoospores are highly sensitive to the chemical environment. For example, a high concentration of salts would trigger the encystment of zoospores, which means that zoospores shed the flagellum and fuse the membrane to form a walled cyst. This can be recognised as a self-protective strategy. But interestingly, even a high concentration of attractants would trigger the encystment of zoospores, which is believed to be the path towards zoospore settlement and parasitism.

Hence, the control of the zoospore environment within the channels would seem to be important and could easily be obtained in the design presented in this work with the adjustment of the attractant solution.

### 3.2. Sensing Results

In preliminary work to confirm that the zoospores could indeed be detected while passing the electrodes and to confirm that the frequency dependence of the signal was roughly as expected from the simulations presented in Figure 3, the zoospores were introduced into the microfluidic channel by a flow generated by the syringe pump, while the in-phase current response to a fixed-frequency (5k–300k Hz) AC signal was monitored in real time.

**Figure 4:**
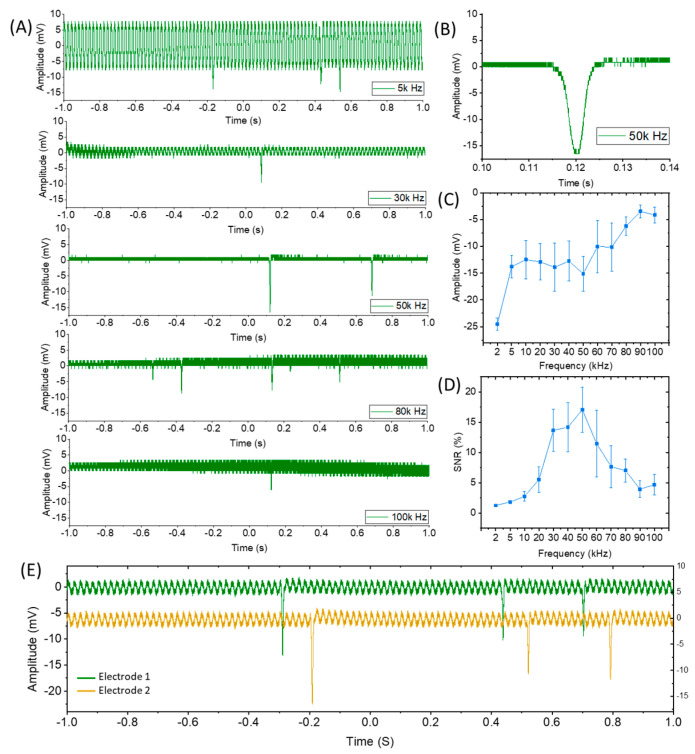
The sensing response from zoospores passing the electrodes, carried in a flow. (**A**) The recorded real-time signal at different AC frequencies. The frequencies presented here are 5 kHz, 30 kHz, 50 kHz, 80 kHz, and 100 kHz from top to bottom. (**B**) A zoomed version of the response recorded at 50 kHz. (**C**,**D**) The statistical analyses of the signal amplitude and signal-to-noise ratio at different frequencies. (**E**) The signal from the two different pairs of electrodes, 1 mm apart in the flow direction.

The passing of zoospores successfully triggered a sharp peak in the recorded signal (Figure 4), which visual observation confirmed was indeed due to the passage of a spore between the electrodes (Supplementary Materials, Video S3). The amplitude and the signal-to-noise ratio (SNR) demonstrated clear dependence on the AC frequency of the input potential sine wave. Either a too-high or too-low frequency would lead to low responses. The statistical analysis of the sensing signal is presented in Figure 4C,D. An optimum SNR was found at around 50 kHz.

Such dependence of the SNR on frequency could be explained together with the equivalent circuit in Figure 3. If the measurement frequency were low, then the impedance would be dominated by the interfacial double-layer capacitance, C_DL_ (Figure 3A). If the measurement frequency were high, then the stray capacitance, C, and the zoospore membrane capacitance, C_M_, would act as short circuits, so the zoospores would be undetectable. At a frequency such that the membrane capacitive impedance was high, the zoospores would cause a change in the conductance of the channel associated with the physical blocking of a part of the channel. The magnitude of this change would depend on the height and width of the channel relative to the diameter of the spore.

Two pairs of electrodes, as two independent sensing channels (CH1 and CH2), were monitored simultaneously. In this way, the travelling speed of the zoospores, which was controlled by the pumping rate, can be calculated as *v* = *L*/*t*, where *v* is the velocity of the zoospores, *L* is the distance between the two pairs of electrodes and *t* is the time difference between CH1 and CH2 when the peak signal appears (Figure 4E). Impressively, by correlating the signal with the optical videos (Supplementary Materials, Video S3), high sensitivity and accuracy in zoospore counting were demonstrated despite the high travelling speed of the zoospores within the flow (~10 mm/s).

**Figure 5:**
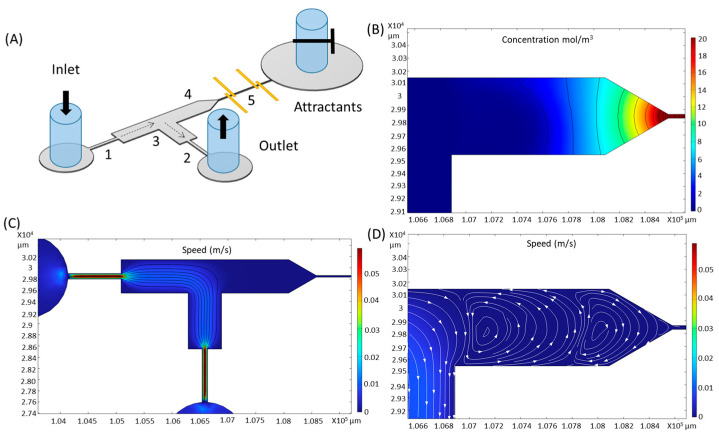
(**A**) Proof-of-concept design for sampling zoospores from a flow. (**B**) The simulation result of the attractant gradient. (**C**) The simulation result of the flow rate distribution inside the microfluidic channel. (**D**) The enlarged flow simulation result showing the eddies into the stub near the entrance of the sensing channel. The numbered features are discussed in the text.

After the above validation using a guiding flow, we utilised chemotaxis as the mechanism for selectively extracting zoospores from the flow, as illustrated in Figure 5A. The idea is to have zoospore samples loaded to one side of the channel and attractants loaded to the other side to form a concentration gradient based on diffusion.

A passive flow was created between the zoospore inlet and the device outlet driven by the height difference of water inside the inlet and outlet tubing. The layout of the channels was rationally designed to facilitate the attraction of zoospores. Firstly, we used narrow ‘necks’ (constrictions (1 and 2)) to restrict the flow rate and then a higher width in the transition zone (3) to slow down the zoospores (Figure 5C), so that the spores could swim across the fluid streamlines. The width of the constraints (1 and 2) is 100 µm, which is significantly larger than the sensing channel (5), thus lowering the risk of channel clogging. Then, an L shape was designed in the transition zone (3) to control the vortex (and hence zoospore) injection into the stub (4). When a stream flows around the bend, which has an indentation at the corner, depending on the angle and shape of the corner and the fluid velocity, a part of the flow moves into the indentation and creates slow-moving eddies within which objects from the flow may be trapped (Figure 5D). The length of the stub (4) controls the number of vortices within the stub and the fluid velocity within them (Figure 5D). A funnel shape was created at the entry of the sensing channel to facilitate the entrance of zoospores. The gradient of the chemotactic attractant within the stub (4) induces zoospores to move across the flow streamlines towards and into the detection channel (5), whose length determines the attractant flux and hence gradient within the stub (4) (Figure 5B).

A sophisticated version of this idea has been demonstrated by Tsai et al. [37]. We have used a simple version for proof of concept. The sampling efficiency of this device is not expected to be high, being determined by the fraction of the total fluid mass flow through the inlet that moves into the vortices within the stub. A device such as that described by Tsai et al., using a sharp angle and a sheath flow to focus the inlet flow into the stub, would be expected to achieve higher sampling efficiency.

The fluid dynamics computation presented in Figure 5 shows a relatively high velocity (>5 cm/s) within the constrictions (1 and 2) when a pressure difference of 100 Pa was applied, slowing to 1 cm/s in the wider part between 1 and 2. A linear gradient of the attractant was created inside the sensing channel (5) (Figure 5B), with a flux that led to the gradient penetrating some distance into the sampling stub (4).

Due to the small dimensions of the microchannel, any small flows or pressure change would cause a major disturbance to the gradient; thus, the initial state of the system would be unknown. To address these issues, we designed two functions in the device: (1) We used a valve on the tubing of the attractant inlet, thus avoiding any passive flow in and out of the sensing channel (channel 5 in Figure 5A). This design also helps avoid the clogging of the sensing channel, as only species that can actively swim could enter the channel. (2) We placed a third channel (zones 3 and 4 and the outlet in Figure 5A) between the zoospore inlet and attractant inlet for creating a controlled and repeatable initial state. Essentially, a syringe pump was used to suck liquid from the outlet after loading water and attractants through the zoospore inlet and attractant inlet, respectively, with the valve on the attractant side open. Due to the different viscosity of the liquids, a phase separation was created at the outlet (Supplementary Materials, Figure S6), which provided us with a known and repeatable initial status. Then, the pumping was slowly stopped and the attractant inlet clamped shut. The two phases mixed with each other and formed a gradient of glucose that, based on the simulation, stabilised within 30 min.

**Figure 6:**
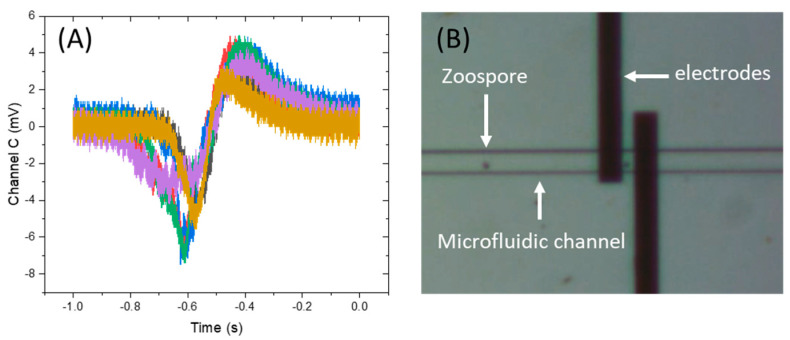
(**A**) Overlap view of sensing signal received from 6 individual zoospores using Tier 2 sensor. Different lines are different individual zoospores. (**B**) Microscope image showing zoospore swimming inside the channel.

A small proportion of the zoospores were carried into the stub (4) and, due to the presence of the attractant gradient, overcame the slow flow within the stub and swam into the sensing channel following the gradient towards a higher concentration. The transit time of the spores through the electrode gap was significantly longer than when they were carried by the pumped flow. The AC coupling differentiated the current pulse, giving a characteristic biphasic signal (Figure 6). A statistical analysis of the data showed the metrics of the sensing signals: amplitude of 10.4 ± 2.0 mV, SNR of 5.9 ± 2.1 and peak-to-peak time of 0.174 ± 0.05 s. The peak-to-peak time is approximately the transit time of the spore across the inter-electrode gap and so indicates a swimming speed of ≈50–100 µm/s. Direct observation confirmed this speed estimate (Supplementary Materials, Video S4).

In the current setup, the visual tracking of zoospores in both the experiments with (Figure 4) and without passive flow (Figure 6) was conducted manually. A relatively low magnification was used, thus allowing for a larger area of view to identify zoospores before they reached the electrodes. Therefore, the quality of the enlarged image is not high, but the presence and passing of zoospores could be clearly seen in Supplementary Materials Videos S3 and S4. This issue could be addressed by using a higher-resolution high-speed camera and motor stages whose movement would be synergistically controlled by automated visual tracking (based on AI and machine learning).

## 4. Discussion

Variations in the signals when using both passive flow (Figure 4) and zoospore actively swimming (Figure 6) could be noticed. This could mainly be attributed to the following two reasons: (1) Natural variation in the size, movement speed and electrical properties of the zoospores in any given population. (2) Even though the minimisation of the dimensions of the sensing channel was performed in the design, there was still space that allowed the zoospores to move up and down, and their Z position was not controlled in this study.

Nonetheless, even though *Phytophthora cactorum* was used as the model zoospore species in this study, our sensing platform has great potential to be further expanded to other zoospore species, including *Phytophthora agathidicida* and even multi-target sensing by using multiple channels.

A glucose solution was used as the attractant in this proof-of-concept study to specifically extract motile zoospores and exclude non-motile interference particles. A higher level of selectivity, to distinguish among different zoospore species, could potentially be achieved by using attracting strategies with a combination of chemical attractants, electrical field, flow track designs and surface properties. The influence of environmental factors, such as temperature, on the behaviour (such as chemotaxis) of zoospores also needs to be taken into consideration for future field devices.

As we have illustrated in Figure 3A, the sensing signals contain important information on the zoospores, including size, membrane capacitance, cytoplasmic resistance and movement speed. Therefore, further analysis of the sensing signals also holds significant potential for distinguishing among different zoospore species. However, this would require multi-frequency measurements, as well as a higher time resolution and a better signal/noise performance of the device.

All these advancements will depend on a deeper understanding of zoospore biology. Our preliminary results are highly promising, demonstrating the ability to sense additional zoospore species and revealing electrotaxis behaviour, where zoospore swimming is significantly influenced by an externally applied electric field. Another exciting direction is enhancing the device with additional functionalities, such as a controlled biomimetic microenvironment, zoospore trapping and in situ individual zoospore analysis. These improvements would transform it into a comprehensive and highly efficient lab-on-a-chip system for zoospore analysis.

The current device is portable, fully battery-powered and capable of data read-out via a laptop. Capabilities such as internal data storage and wireless communication would be useful. Such devices could be applied in the field by, for example, integrating them with a groundwater circulating/collecting system or by combining them with zoospore baits.

## 5. Conclusions

We have developed a microfluidic biosensor for the detection of motile pathogen-related zoospores. Finite element simulation using COMSOL provided an in-depth understanding of the electrical field, passive flow and the diffusion of chemicals, which was helpful in guiding the design of these systems. In our validation assays using a flow to carry zoospores into the sensing channel, high sampling efficiency and fast response in zoospore sensing were demonstrated. After incorporating the chemotaxis function into the microfluidic device, the sensor successfully attracted zoospores to actively swim into the channel. A good sensing response with high signal-to-noise ratio was demonstrated. The device fulfilled its design criteria, successfully sampling and counting motile zoospores. This work has the potential to be further developed into a portable and effective field-based tool to support field-based surveillance. Such devices are critical to real-time detection and can be readily integrated into existing pathogen surveillance and pest management strategies.

## Data Availability

All data related to this paper are presented in the paper and/or the Supplementary Materials.

## Supplementary Materials

The following supporting information can be downloaded at: https://www.mdpi.com/article/10.3390/bios15030131/s1, File S1, Figure S1: Schematic of the microfluidic chip fabrication. (A) Photolithography of the Au electrodes. (B) Fabrication of the PDMS microfluidic channel. (C) Schematic of the assembled device.; Figure S2: (A,B) Optical microscope images of the paired Au electrodes and one example of PDMS microchannels, respectively. (C) The top view optical profiler data of the PDMS channel. Different colours correspond to different heights. (D,E) Height information along the *X*- and *Y*-axes, respectively, as marked in (C); Figure S3: (A) Schematic illustration of attractant testing setup. (B,C) Microscope images of well without and with attractant (1 wt% glucose), respectively. The grey dots are individual zoospores; Figure S4: A photo showing the pogo pin PCB connector (left), the lock-in amplifier (right) and the connection cables; Figure S5: The COMSOL simulation of the sensing response with different (A) channel widths and (B) gap distances between the paired Au electrodes; Figure S6: Optical microscope images showing the phase separation of two liquids during filling; Video S1: Release of *Phytophthora cactorum* zoospores; Video S2: Chemotaxis of *Phytophthora cactorum* zoospores; Video S3: Zoospore sensing in pumping setup; Video S4: Zoospore sensing with them swimming through the channel under the gradient of attractant.
