# Supplementary material for: Microfluidic Biosensors for the Detection of Motile Plant Zoospores
Source: Biosensors (Basel). 2025 Feb 21;15(3):131. doi: 10.3390/bios15030131 (PMC11940179; doi:10.3390/bios15030131)
Supplement: Supplementary file 1 [file biosensors-15-00131-s001.zip › Supporting File S1.docx]

**Supporting Information for**

**Microfluidic Sensors for the Detection of Motile Plant Zoospores**

**Peikai Zhang ^1-3^, David E. Williams ^1,3,^*, Logan Stephens ^4^, Robert Helps ^4^, Shamini Pushparajah ^5^, Jadranka Travas-Sejdic ^1,3^* and Marion Woode ^5,^***

^1^ Centre for Innovative Materials for Health, School of Chemical Sciences, The University of Auckland, Auckland 1010, New Zealand;

^2^ Auckland Bioengineering Institute, The University of Auckland, Auckland 1010, New Zealand;

^3^ MacDiarmid Institute for Advanced Materials and Nanotechnology, Wellington 6140, New Zealand;

^4^ inFact Limited, Christchurch 8013, New Zealand;

^5^ The New Zealand Institute for Plant & Food Research Limited, Auckland 1025, New Zealand;

***** Correspondence: e-mail@e-mail.com; david.williams@auckland.ac.nz (D. E. W.); j.travas-sejdic@auckland.ac.nz (J. T. S.); marion.wood@plantandfood.co.nz (M. W.)

Contents

S1. Microfluidic chip fabrication

S2. *Phytophthora cactorum* zoospore production

S3. Chemotaxis of zoospores

S4. Electronic design and recording setup

S5. Simulation results for various channel heights and electrode spacings

S6. Phase separation during channel filling

Video S1: Release of Phytophthora cactorum zoospores

Video S2: Chemotaxis of Phytophthora cactorum zoospores

Video S3: Zoospore sensing in the pumping setup

Video S4: Zoospore sensing with them swimming through the channel under the gradient of attractant

S1. Microfluidic chip fabrication

The microfluidic chips, including both the PDMS microchannel and the Au microelectrodes (**Figure S1**), were fabricated via the standard photolithography technique in a cleanroom environment.

The Au electrodes were patterned with Au- (100 nm) and Ti-coated (40 nm) glass substrates (Deposition Research Lab, Inc, Saint Charles, MOI, USA) via photolithography. Briefly, a thin layer of AZ nLOF 2070 negative photoresist (MicroChemicals, Ulm, Germany) was patterned by ultraviolet (UV) light through a shadow mask on top. The patterned photoresist acted as a protective layer for the subsequent chemical etching steps. The unprotected Au and Ti were etched away by a KI/I_2_ (10 wt% KI and 2.5 wt% I_2_) solution and a HCl (50 vol% in water; 65 ^o^C) solution, respectively. After this, acetone and isopropyl alcohol were used to rinse the Au electrodes to remove the photoresist. The Au electrodes were then dried with N_2_. The width of each Au electrode was 50 µm, and the spacing between the two was 10 µm. There were two pairs of sensing electrodes inside each microfluidic channel. The distance between these two pairs was 1000 µm.


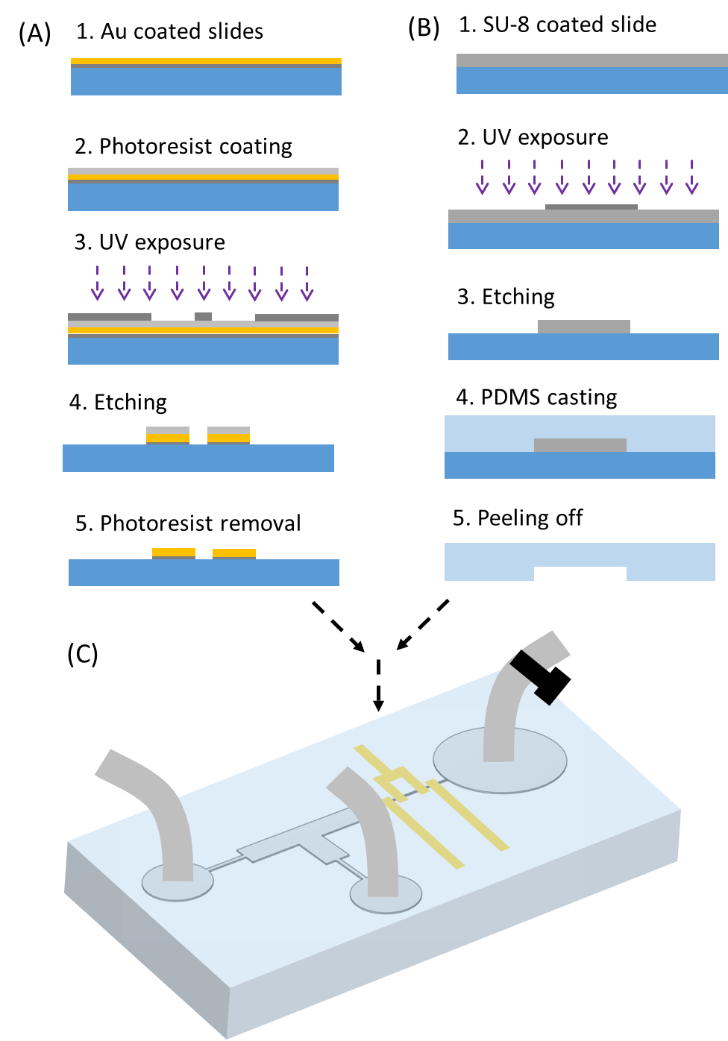


Figure. S1. Schematic of the microfluidic chip fabrication. (A) Photolithography of the Au electrodes. (B) Fabrication of the PDMS microfluidic channel. (C) Schematic of the assembled device.

For the SU-8 mould, a 20 µm layer of the negative photoresist, SU-8 (KayakuAdvanced Materials, Westborough, MA, USA), was spin-coated on an O_2_ plasma-treated glass substrate. This SU-8 layer was then patterned via photolithography using a shadow mask and etched with propylene glycol monomethyl ether acetate. The SU-8 mould was finally rinsed with isopropyl alcohol and dried with N_2_. Poly(dimethylsiloxane) (PDMS; Sylgard 184, Dow, Midland, MI, USA) was prepared by mixing the monomer and curing agent in a 10:1 weight ratio, which was subsequently vacuumed to remove the bubbles and cast onto the SU-8 mould. After being baked at 80 ^o^C for 2 hours, the PDMS solidified. It was then peeled off from the mould and cut into parts with suitable sizes and replicated microchannel structures. The height and width of the microfluidic channels were characterised by an optical profilometer (ContourX-100, Bruker, Billerica, MA, USA) and presented in **Figure S2**.


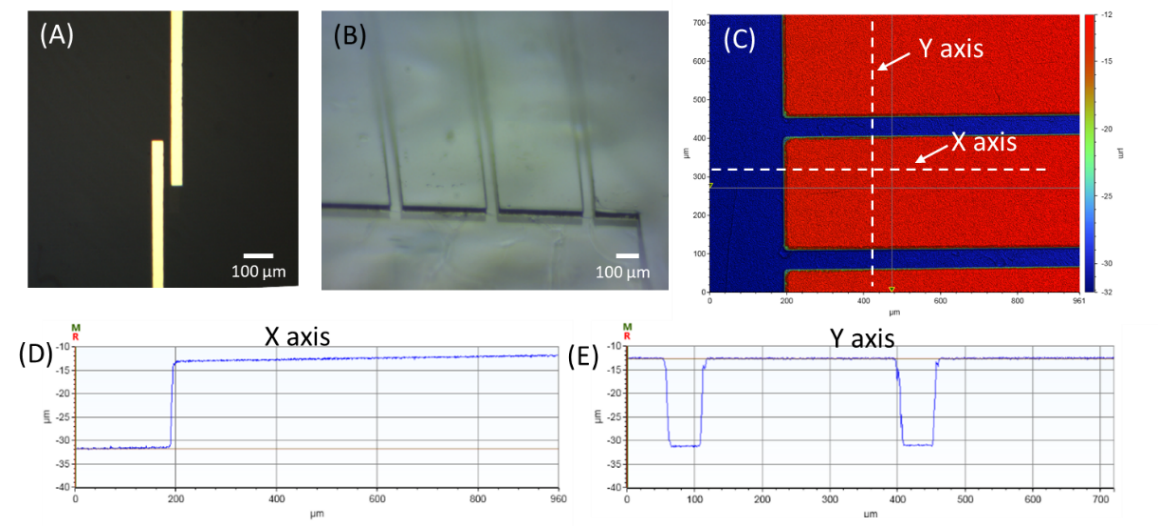


Figure. S2. (A) and (B) Optical microscope images of the paired Au electrodes and one example of PDMS microchannels, respectively. (C) The top view optical profiler data of the PDMS channel. Different colours correspond to different heights. (D) and (E) Height information along the X- and Y-axes, respectively, as marked in (C).

S2. Phytophthora cactorum zoospore production

Due to the pathogen *Phytophthora agathidicida* being classed as an unwanted organism (UWO) as defined by the New Zealand Biosecurity Act 1993, its use in these experiments was not possible. As a relevant model system, an alternative Phytophthora species, *Phytophthora cactorum*, was chosen.

**S2.1 Preparation of *Phytophthora cactorum* culture plates**

By using a sterile hood and standard sterile operating techniques, *Phytophthora cactorum* culture H78 (original plate provided by Dr. Ian Horner PFR) was plated out by using a 5 mm x 5 mm plug from the original plate onto a fresh 10 % V8 agar plate (see Laboratory Protocols for *Phytophthora* Species Protocol 07-11.1 (November 2015) http://dx.doi.org/10.1094/9780890544969.07.11.1). Plates were incubated at room temperature for 5 days until visible mycelial growth. Then, 5 mm x 5 mm plugs were excised from the margins of visible growth and placed into sterile McCartney bottles containing 30 mL of sterile MilliQ water. The McCartney bottles were stored at 5-10 ^o^C.

**S2.2 *Phytophthora cactorum* oat inoculation**

A total of 100 g of oats (*Avena sativa*; PGG Wrightson Grain’s Gusto Range Oats, New Zealand) was placed in a 500 mL Schott bottle containing 300 mL of MilliQ water for 24 hr at room temperature. The water was removed, and the hydrated oats were sterilised for 20 min at 121 ^o^C with one additional repeat. Once cooled, the oats were inoculated with 15 5 mm x 5 mm agar plugs cut from the edges of freshly gown *Phytophthora cactorum* mycelial growth by using sterile operating techniques. The oats were gently agitated and the lids secured. The oat/*Phytophthora cactorum* mix was placed at room temperature in the dark for 7-10 days with gentle agitation every 2 days to ensure even colonization. Once colonialized, the oat/*Phytophthora cactorum* mix was stored at room temperature in the dark.

**S2.3 Preparation of *Phytophthora cactorum* zoospores**

By using a sterile hood and standard sterile operating techniques, 15 oats were carefully removed from the oat/*Phytophthora cactorum* mix and placed in a sterile Petri dish containing 20 mL of sterile water to induce sporangium formation. The Petri dish lid was replaced, and the colonized oats were placed under standard long-day growth conditions (16 hr light / 8 hr darkness) for 3 - 4 days. To induce zoospore release, the Petri dish was placed at 4 ^o^C for 45 min followed by 30 - 45 min incubation at room temperature in the light. Zoospore production was monitored by using a Nikon Eclipse Ni microscope at 10X magnification under standard bright-field observation. The concentration of the zoospore solution was adjusted by using sterile water where necessary, and the zoospore preparations were discarded after 8 hr.

S3. Chemotaxis of zoospores

The experimental setup of the chemotaxis study and the microscope images showing the different populations of zoospores from the medium with and without the attractant are presented in **Figure S3.**


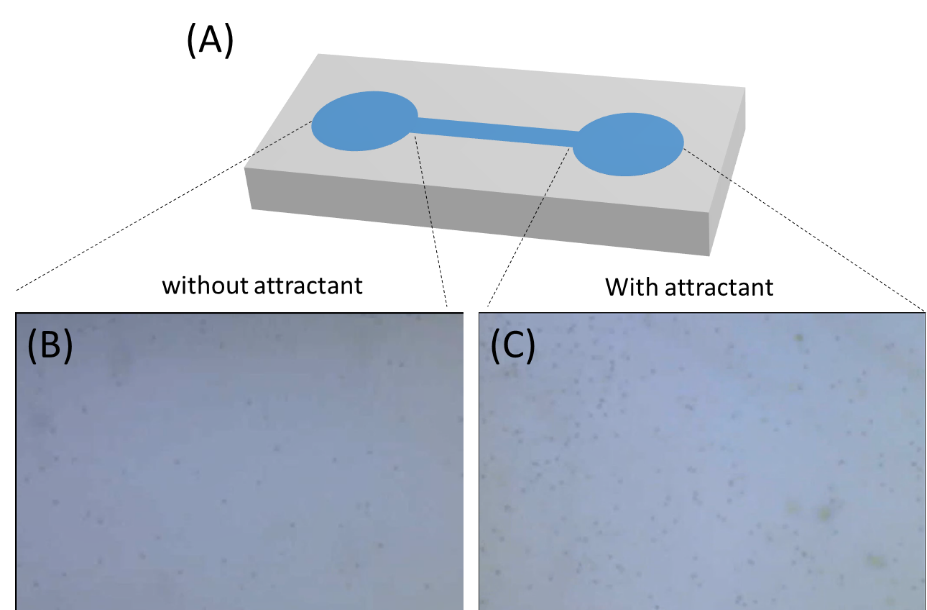


Figure. S3. (A) Schematic illustration of attractant testing setup. (B) and (C) Microscope images of well without and with attractant (1 wt% glucose), respectively. The grey dots are individual zoospores.

# S4. Electronic design and recording setup

A Printed Circuit Board (PCB) with pogo pin connectors was designed and fabricated by inFact for connecting to the microfluidic sensor (**Figure S4**). An observation opening was left in the middle of the PCB to enable the penetration of light through the microfluidic chip for optical observation with the microscope. The PCB connector was then connected to an oscilloscope (Picoscope 2000) through a lock-in amplifier for the waveform AC input and signal recording. The lock-in amplifier rejects noise outside the frequency of interest, enabling the detection of the small changes caused by passing zoospores.

A camera with microscopic lens was placed above the sensing chip, while LED light was placed under the chip. The optical video was recorded simultaneously with the electrical recording; thus, the sensing signal could be correlated with the optical information.


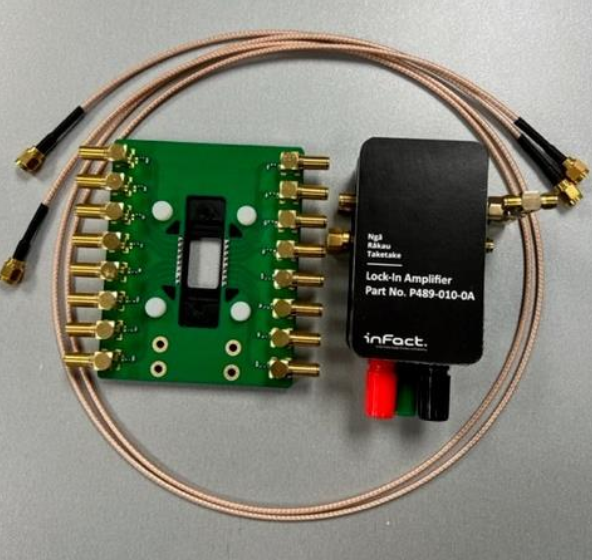


Figure. S4. A photo showing the pogo pin PCB connector (left), the lock-in amplifier (right) and the connection cables.

S5. COMSOL Simulation

The COMSOL simulation results of the sensing response for various channel heights (10-50 µm) and electrode spacings (5-50 µm) are shown in **Figure S5**.


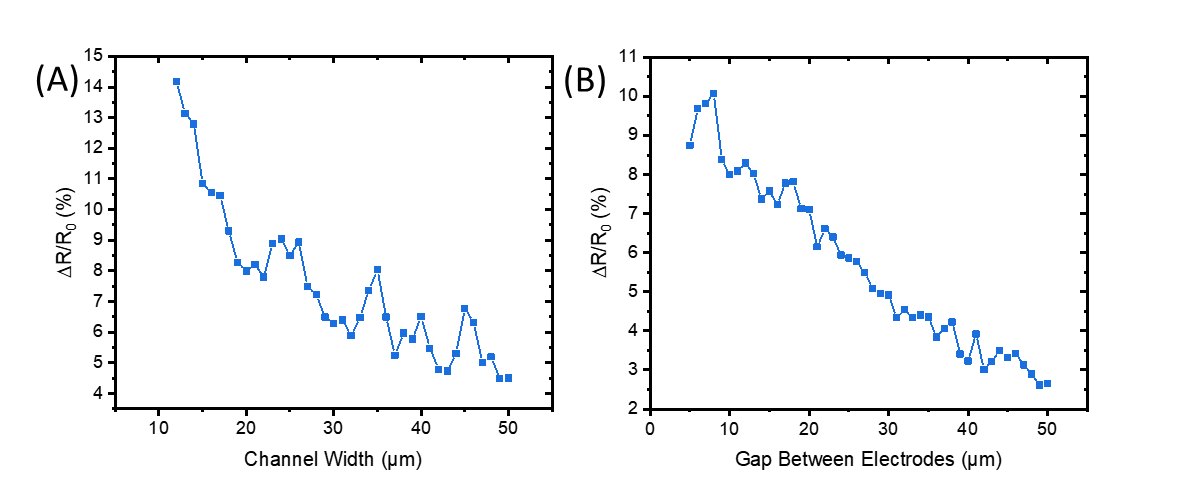


Figure. S5. The COMSOL simulation of the sensing response with different (A) channel widths and (B) gap distances between the paired Au electrodes.

S6. Channel filling

The phase separation of the two liquids (water and 1 wt% glucose) during the filling of the microfluidic chip is shown in **Figure S6**. A yellow dye was added to the glucose solution for a better visual effect.


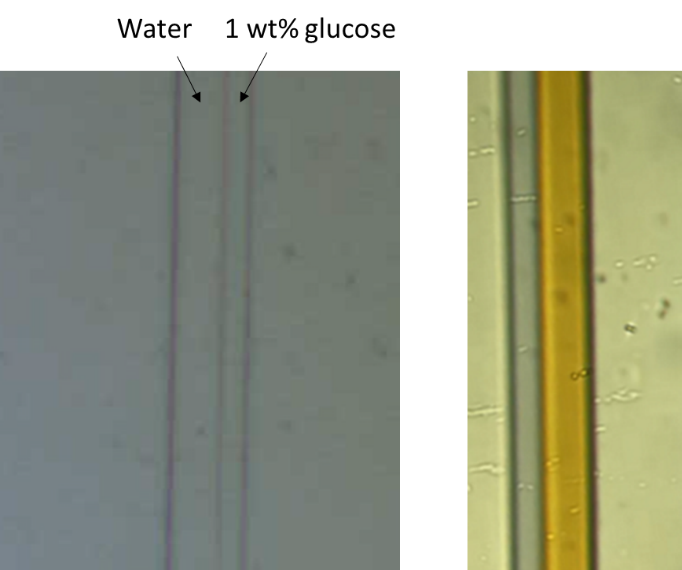


Figure. S6. Optical microscope images showing the phase separation of two liquids during filling.
